# Current status and future perspectives of contrast-enhanced ultrasound diagnosis of breast lesions

**DOI:** 10.1007/s10396-024-01486-0

**Published:** 2024-08-23

**Authors:** Toshikazu Ito, Hironobu Manabe, Michiyo Kubota, Yoshifumi Komoike

**Affiliations:** Division of Breast and Endocrine Surgery and Department of Surgery, Kindai University Faculty of Medicine, Osaka, Japan

**Keywords:** Breast lesions, Ultrasound, Contrast-enhanced ultrasound (CEUS), Sonazoid, Liver

## Abstract

Advances in various imaging modalities for breast lesions have improved diagnostic capabilities not only for tumors but also for non-tumorous lesions. Contrast-enhanced ultrasound (CEUS) plays a crucial role not only in the differential diagnosis of breast lesions, identification of sentinel lymph nodes, and diagnosis of lymph node metastasis but also in assessing the therapeutic effects of neoadjuvant chemotherapy (NAC). In CEUS, two image interpretation approaches, i.e., qualitative analysis and quantitative analysis, are employed and applied in various clinical settings. In this paper, we review CEUS for breast lesions, including its various applications.

## Introduction

In recent years, with the inclusion of testing for hereditary breast and ovarian cancer syndrome (HBOC) under insurance coverage, there has been an increase in the number of patients diagnosed with HBOC. The cumulative incidence risk of breast cancer by age 80 is reported to be 72% for *BRCA1* mutation carriers and 69% for *BRCA2* mutation carriers, while the cumulative incidence risk of ovarian cancer is 44% for *BRCA1* and 17% for *BRCA2* [1]. Magnetic resonance imaging (MRI) screening is recommended for follow-up of *BRCA*-positive breast cancer patients and screening of asymptomatic individuals [2], yet the inability of certain patients to undergo MRI for various reasons remains problematic. Triple-negative breast cancer is prevalent in *BRCA1* mutation carriers, whereas estrogen receptor-positive, HER2-negative luminal breast cancer is more common in *BRCA2* mutation carriers [3]. While mammography detects calcifications in *BRCA2*-related breast cancer, reports indicate a lack of calcifications in *BRCA1*-related breast cancer [4, 5]. Due to the difficulty in diagnosing HBOC-related breast cancer using mammography or conventional ultrasound (US), there is a potential increase in the importance of contrast-enhanced US (CEUS) as an alternative imaging modality to MRI [6]. CEUS, being capable of clearly demonstrating the microvascular perfusion within and around tumors, represents an innovative diagnostic technique allowing for more accurate real-time evaluation of microvascular structures of breast lesions [7–9]. Additionally, CEUS can depict small vessels that may not be detected on MRI [10].

In CEUS, either qualitative analysis through contrast-enhanced pattern analysis or quantitative analysis through contrast-enhanced kinetic analysis, or both, are employed for differentiation between benign and malignant lesions [8]. CEUS proves beneficial in various aspects of breast cancer management, including differential diagnosis of breast lesions, assessment of tumor spread, staging of invasive cancer, evaluation of the effectiveness of neoadjuvant chemotherapy (NAC), and diagnosis of axillary lymph node metastasis.

In recent years, the availability of new therapeutic agents tailored to the biology and intrinsic subtype of breast cancer, as evidenced by clinical trial results, has led to a diversified treatment approach, necessitating personalized treatment strategies. Selection of available therapeutic agents is determined based on pre-treatment evaluations such as the recurrence score derived from the 21-gene breast cancer assay and the number of metastatic lymph nodes, even within the same intrinsic subtype of breast cancer [11, 12]. Evaluation of lesions, including lymph node metastasis status, is crucial across all patient groups, and accurate determination of treatment strategies through CEUS-based assessment and diagnosis may lead to improved treatment outcomes. The aim of this review is to discuss the various utilities of CEUS with the objective of elucidating its effectiveness.

## History of CEUS

Contrast-enhanced methods in US imaging had their origins in 1969 when Gramiak et al. first utilized indocyanine green (ICG) as a contrast agent in cardiovascular US imaging [13]. By the late 1970s, it was discovered that the source of echo enhancement in CEUS was microbubbles [14]. In 1982, Matsuda et al. initiated CEUS of liver tumors using the CO_2_ microbubble injection method, establishing diagnostic criteria and paving the way for its widespread adoption [15–19]. Subsequently, the availability of Levovist, a contrast agent suitable for intravenous administration at high mechanical index (MI) values, became feasible [20]. Furthermore, the development of second-generation contrast agents such as Optison, Definity, SonoVue, and Sonazoid [21–25] enabled visualization of blood flow signals at low to moderate MI values, expanding the application of CEUS to areas including the liver, biliary tract, pancreas, and breast regions.

## Differences between CEUS in the liver and breast regions

The liver is characterized by a dual vascular supply from the artery and portal vein, resulting in two distinct vascular phases: the arterial-dominant phase and the portal-dominant phase. Moreover, in CEUS using Sonazoid, unlike other contrast agents such as Definity or SonoVue, Kupffer cells in the liver’s reticuloendothelial system uptake Sonazoid, resulting in the presence of two contrast enhancement phases: the vascular phase and the post-vascular phase (Kupffer phase, typically observed 10 min after contrast agent injection) [26–28].

While blood flow in normal liver tissue is abundant, it is relatively less so in normal breast tissue. However, relatively strong enhancement effects may be observed in premenopausal breast tissue. Therefore, in contrast-enhanced MRI, it is recommended to consider the menstrual cycle and perform imaging between days 5 and 12 after the onset of menstruation. Due to the relatively deeper imaging depth in the liver, probes with frequencies around 3.5 MHz are used, whereas for breast imaging, probes with relatively higher frequencies are utilized as lesions may be present up to 3–5 cm deep under the skin.

**Fig. 1:**
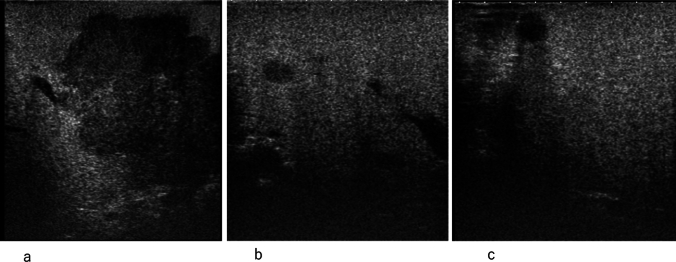
Metastatic liver tumors. **a**, **b**, **c**, Sonazoid contrast-enhanced ultrasound enables the detection of small malignant liver lesions

Advances in CEUS imaging, coupled with the development of second-generation contrast agents, have endowed CEUS with the ability to sensitively and accurately depict tumor vasculature. Consequently, significant improvements have been achieved in the diagnosis of focal liver lesions, including hepatocellular carcinoma (HCC) [29]. By utilizing Sonazoid, which does not contain Kupffer cells, the diagnostic sensitivity for malignant liver tumors is increased. Sonazoid CEUS enables the detection of small malignant liver lesions, including metastatic liver tumors, determination of the surgical approach for liver resection, appropriate guidance for non-surgical ablation techniques such as radiofrequency ablation, and accurate evaluation of treatment response to drug therapies, including those for HCC [30–32] (Fig. 1).

In the Kupffer phase of Sonazoid CEUS, it is possible to scan the entire liver, allowing for the detection of HCC even when lesions are relatively small, making it valuable for HCC surveillance [29–32]. Furthermore, utilizing reperfusion techniques involving reinjection of contrast agents enables the diagnosis of newly detected lesions in the Kupffer phase [30].

## CEUS Diagnostic criteria for breast lesions

### Contrast agents

When using SonoVue as the contrast agent, imaging is typically performed at a low MI value, generally around 0.06–0.08. On the other hand, when using Sonazoid, imaging is usually conducted at a moderate MI value, typically around 0.2. SonoVue is composed of sulfur hexafluoride and is a stable aqueous suspension of microbubbles encapsulated by a lipid shell [33]. Sonazoid, on the other hand, is considered to exhibit higher stability than SonoVue due to its stable outer shell containing hydrogenated egg phosphatidylserine, which enables it to withstand pressure and minimize bubble collapse and signal loss [22, 33, 34].

The gas in microbubbles can pass through pulmonary capillary filters and be exhaled through lung respiration. Long-term safety has been confirmed in the liver and breast regions [21, 24, 25, 35]. The size of second-generation contrast agents is comparable to that of red blood cells, preventing them from passing through the vessel wall into the interstitial space. As a result, these contrast agents can directly and accurately reflect the microcirculation perfusion of lesions, thereby improving the diagnostic agreement rate of breast lesions [36].

### Qualitative diagnostic evaluation

**Fig. 2:**
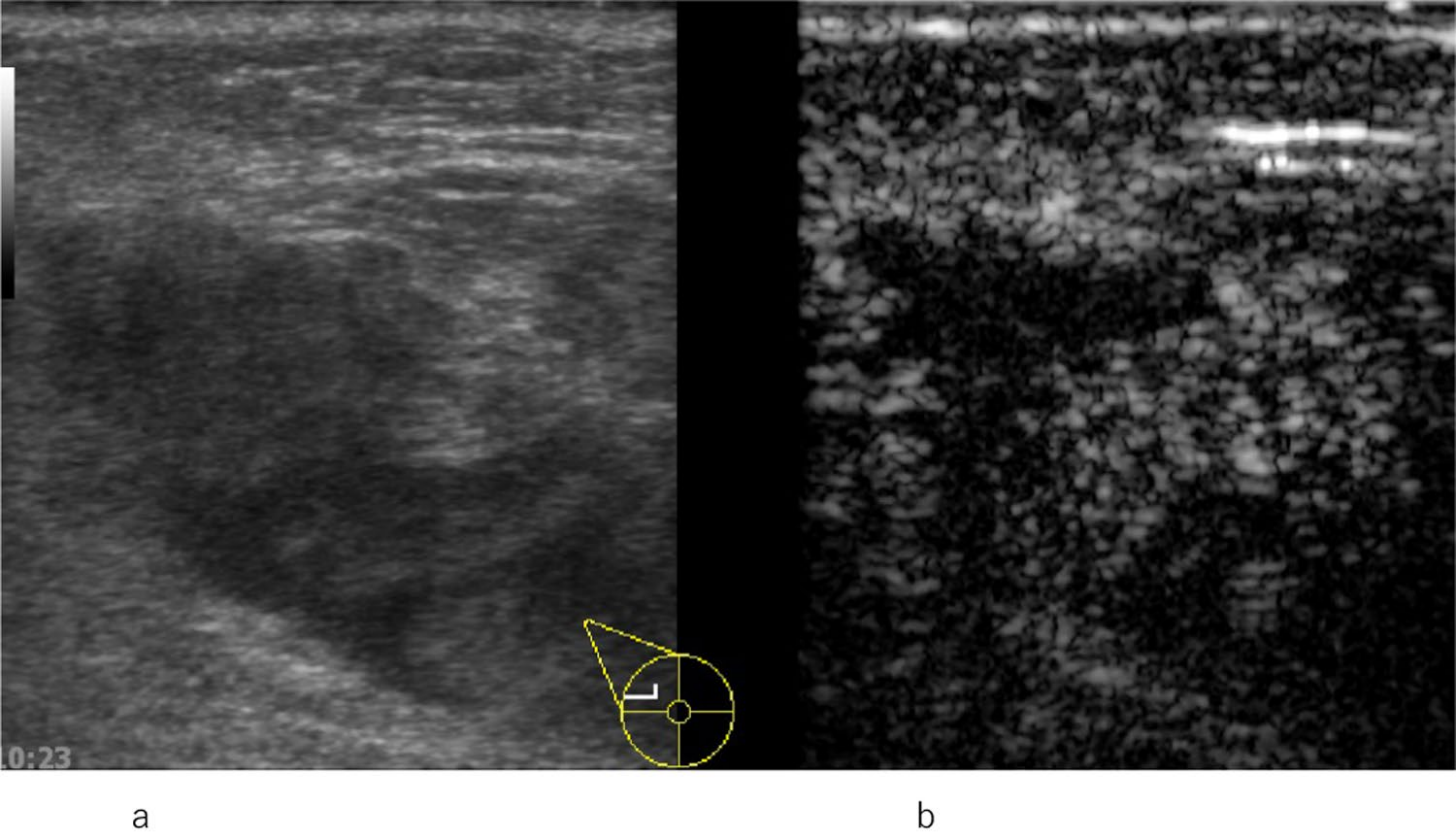
Invasive ductal carcinoma. **a** Conventional ultrasound shows a hypoechoic area. **b** Contrast-enhanced ultrasound shows heterogeneous enhancement with a clear defect

**Fig. 3:**
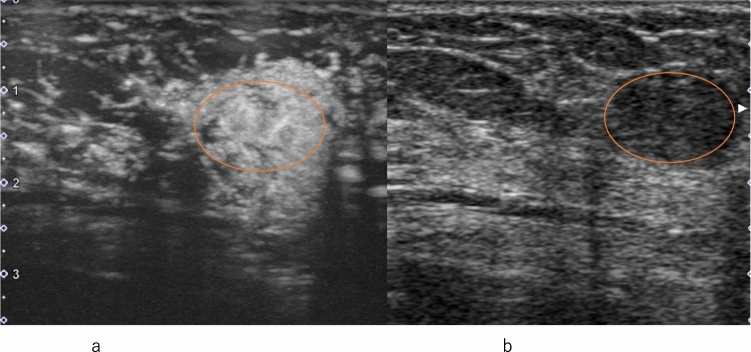
Invasive ductal carcinoma. **a** Contrast-enhanced ultrasound shows heterogeneous enhancement without a clear defect (circle). **b** Conventional ultrasound shows a hypoechoic area (circle)

**Fig. 4:**
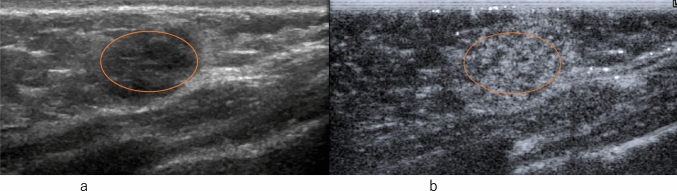
Invasive ductal carcinoma. **a** Conventional ultrasound shows a hypoechoic area (circle). **b** Contrast-enhanced ultrasound shows heterogeneous enhancement extending outward beyond the expected border of the lesion (circle)

**Fig. 5:**
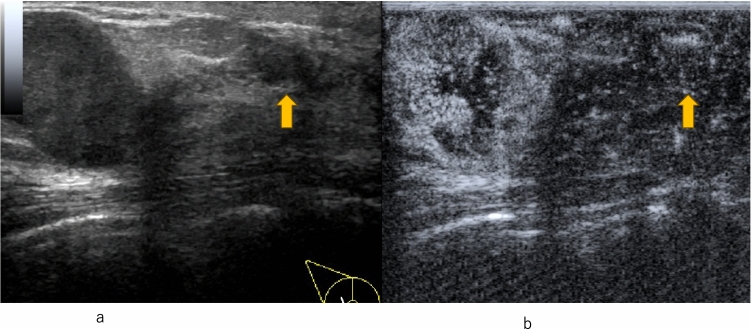
Invasive ductal carcinoma. **a** Conventional ultrasound shows a mass with a small hypoechoic daughter lesion (arrow). **b** Contrast-enhanced ultrasound of a small hypoechoic daughter lesion (arrow) shows heterogeneous enhancement

**Fig. 6:**
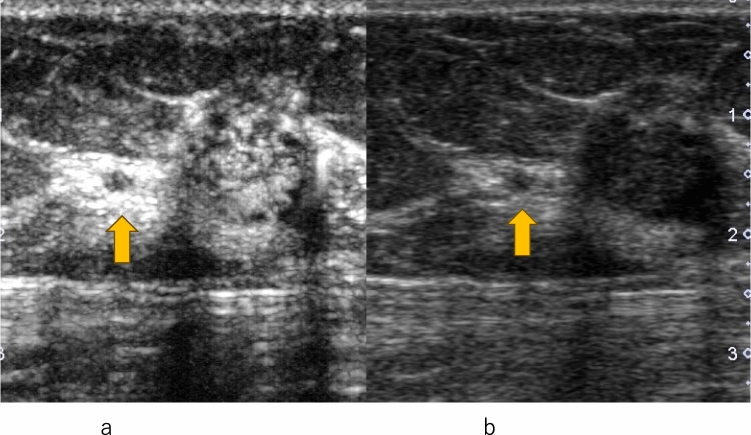
Invasive ductal carcinoma. **a** Contrast-enhanced ultrasound of a small hypoechoic daughter lesion (arrow) shows heterogeneous enhancement. **b** Conventional ultrasound shows a mass with a small hypoechoic daughter lesion (arrow)

The following diagnostic criteria have been utilized in clinical trials of Sonazoid CEUS for breast lesions and have been widely adopted clinically due to their relatively straightforward application and favorable diagnostic performance [25]:

Enhancement patterns indicative of benign lesions include strong or homogeneous enhancement of the entire lesion, or lack of enhancement of the entire lesion. Enhancement patterns indicative of malignant lesions include heterogeneous enhancement with or without clear defects (Fig. 2, Fig. 3), rapid washout from the lesion compared to washout from the surrounding mammary tissues, and the degree of enhancement being greater than that of the surrounding tissue, and the area of enhancement being larger than the hypoechoic lesion on the precontrast (or conventional US) images (Fig. 4, 5, 6).

Several scoring systems and qualitative evaluation criteria have been proposed, generally focusing on similar evaluation parameters. Specifically, benign lesions typically exhibit synchronous or delayed enhancement, homogeneous or low enhancement, clear margins, and regular shapes, whereas malignant lesions often show early heterogeneous over-enhancement, indistinct margins, irregular shapes, and an expanded area of enhancement compared to conventional US tumor size [37–39]. Regarding expansion of tumor size, it is defined as enlargement when either the length or width increases by more than 3 mm compared to conventional US measurements [40].

**Fig. 7:**
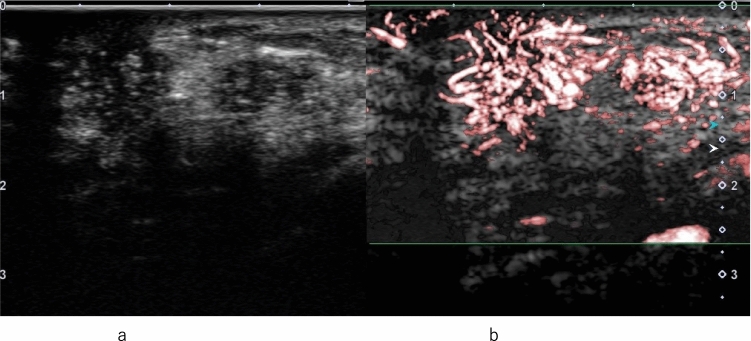
Invasive ductal carcinoma. **a** Conventional ultrasound shows a hypoechoic area. **b** Contrast-enhanced ultrasound shows heterogeneous enhancement with a clear defect extending outward beyond the expected border of the lesion

Other findings indicative of malignant lesions through qualitative evaluation include heterogeneous and centripetal enhancement, as well as the presence of peripheral radial or penetrating vessels, whereas benign lesions predominantly demonstrate homogeneous and centrifugal enhancement [8]. Regarding the characteristics of vascular architecture assessed qualitatively, malignant lesions typically exhibit tortuous and irregular vessels, whereas benign lesions show gently curved vessels along the tumor margins. Arteriovenous shunting is observed in malignant lesions but not in benign lesions [41, 42]. Additionally, malignant lesions often show heterogeneous distribution of vessels and frequently exhibit local perfusion defects [8, 41–43] (Fig. 7).

### Quantitative diagnostic evaluation

Quantitative parameters obtained from the time-intensity curve (TIC) include the following: peak intensity (%), which is defined as the maximum intensity value in the time-intensity curve; time-to-peak (TTP) (sec), defined as the duration between the moment when the contrast medium first reaches the lesion and the time of maximum signal intensity after contrast medium administration; mean transit time (MTT) (sec), defined as the duration of enhancement of the lesion; regional blood volume (RBV) (mL), which represents the area under the TIC, reflecting the total volume of contrast medium passing through the lesion of interest; and regional blood flow (RBF) (mL/sec), calculated as the fraction area under the curve divided by MTT, reflecting the relative blood flow in the selected lesion’s area [44].

Regarding quantitative parameters, malignant lesions typically exhibit significantly shorter TTP, higher peak intensity, and increased wash-in slope. While the TICs of malignant lesions are predominantly plateau and wash-out types, benign lesions mainly demonstrate plateau and slow-rise types [39, 45]. Factors involved in the differentiation between benign and malignant lesions using CEUS with Sonazoid include not only enhancement patterns but also the slope of the tangent at the starting point of the TIC (Axk value), as demonstrated by Fujimitsu et al. [46]. The Axk value is defined as the slope of the tangent at the beginning of the TIC.

## Characteristics of CEUS in non-mass abnormalities

**Fig. 8:**
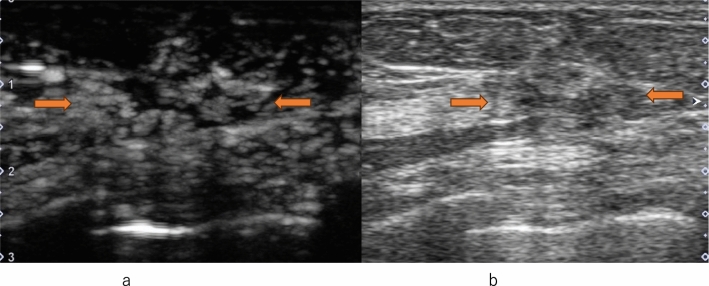
Ductal carcinoma in situ. **a** Contrast-enhanced ultrasound of a small hypoechoic lesion (between arrows) shows heterogeneous enhancement. **b** Conventional ultrasound shows a hypoechoic area of a non-mass abnormality (between arrows)

**Fig. 9:**
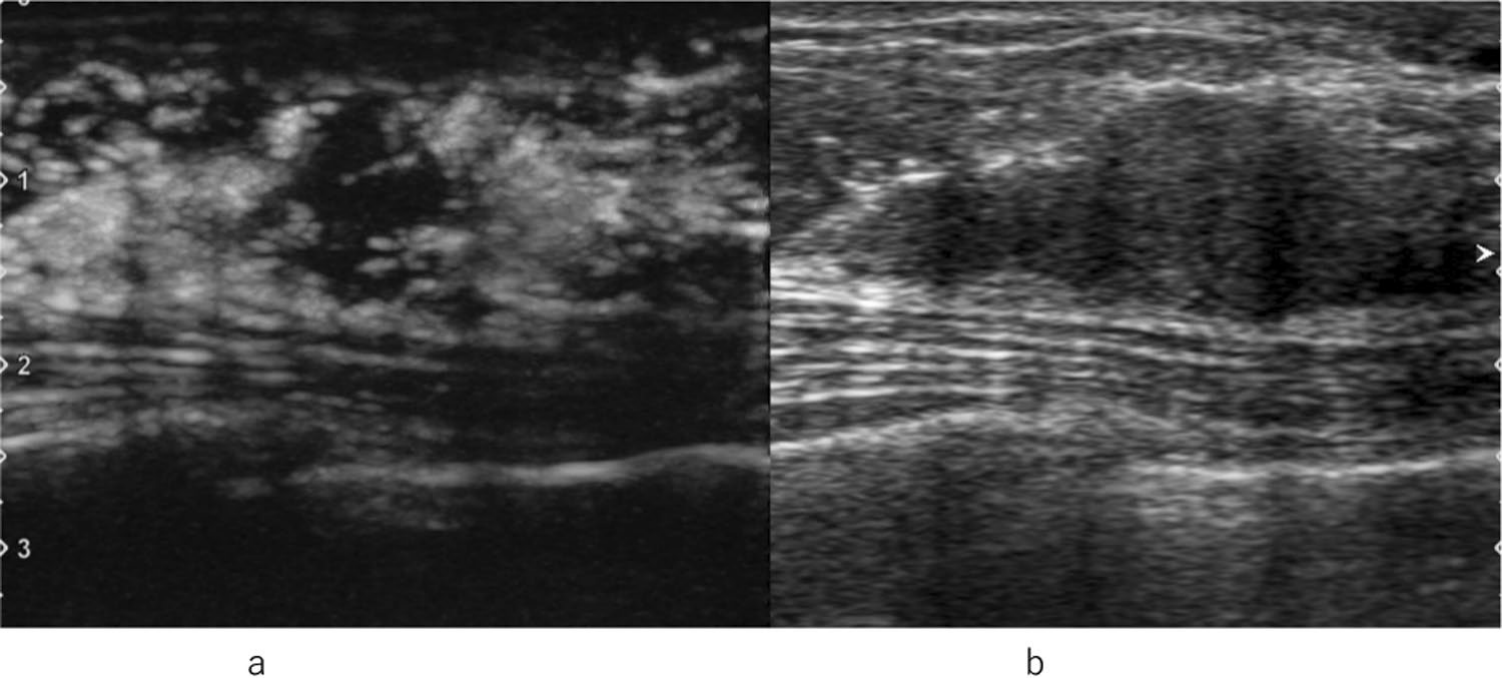
Invasive ductal carcinoma. **a** Contrast-enhanced ultrasound shows heterogeneous enhancement with a clear defect. **b** Conventional ultrasound shows a hypoechoic area

**Fig. 10:**
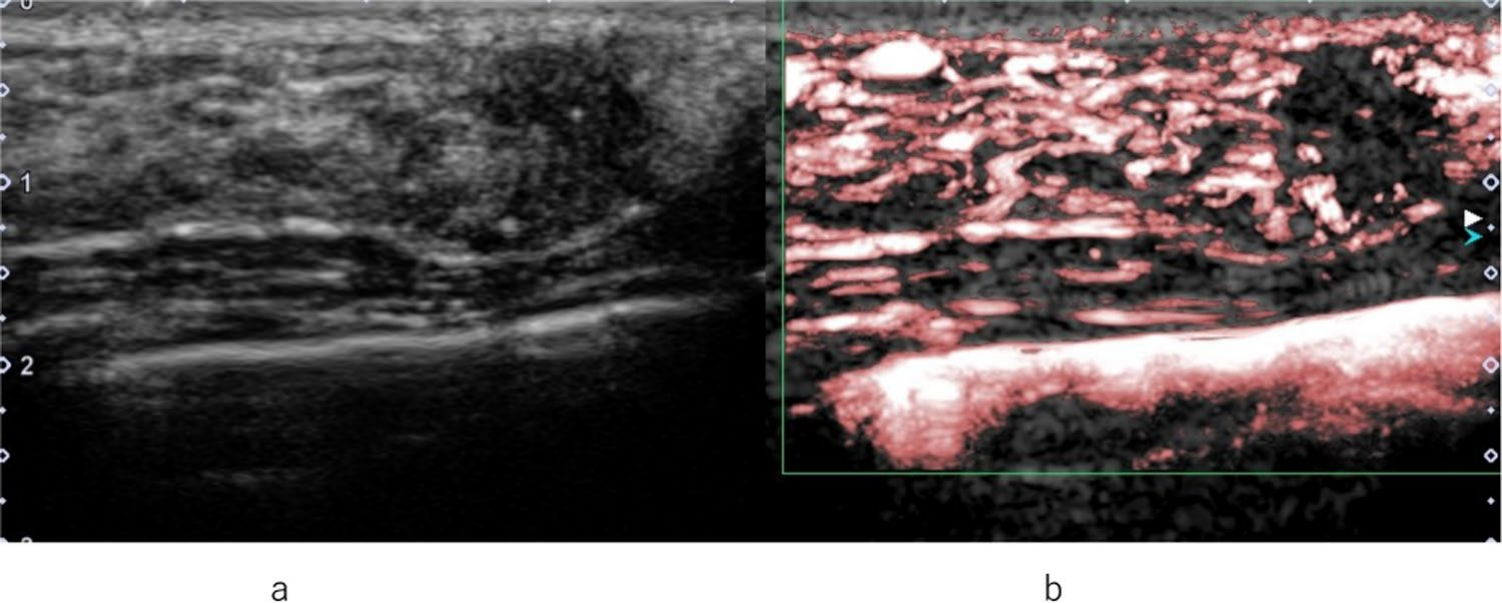
Ductal carcinoma in situ. **a** Conventional ultrasound shows a hypoechoic area in the mammary gland with echogenic foci. **b** Contrast-enhanced ultrasound shows heterogeneous enhancement with a clear defect

With respect to terminology related to non-mass abnormalities, other terms such as non-mass lesions, non-mass-like lesions, and non-mass breast lesions may be used interchangeably with similar definitions. Guidelines for non-mass abnormalities (NMAs) in conventional US were established by the Japan Society of Ultrasonics in Medicine in 2023, and several review articles have been published [47–53]. However, reports on the diagnosis of NMAs using CEUS are currently limited [54–56]. Malignant NMAs are characterized by early wash-in time, hyper-enhancement degree, unclear enhancement margin, enlarged enhancement area, and early wash-out time. Conversely, benign NMAs are primarily characterized by the absence of radial or penetrating vessels and perfusion defects [45, 56, 57]. In non-mass abnormalities, CEUS can clearly demonstrate the area of the lesion and the status of internal vessels, enabling accurate guidance for biopsy sites [38]. Factors predicting malignant ductal lesions among non-mass abnormalities include the presence of microcalcifications and enlargement of the enhancement area [58] (Figs. 8, 9,10).

## Characteristics of CEUS in pathological prognostic factors

**Fig. 11:**
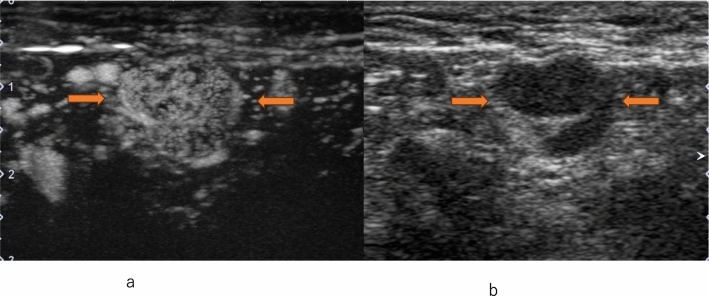
Metastatic lymph node. **a** Contrast-enhanced ultrasound shows heterogeneous enhancement (between arrows). **b** Conventional ultrasound shows an axillary lymph node with cortical thickening (between arrows)

The majority of HER2-positive breast cancers exhibit heterogeneous enhancement, while ER-negative breast cancers often demonstrate centripetal enhancement. Perfusion defects are frequently observed in cancers with high malignancy grades such as HER2-positive, ER-negative, and Ki-67-positive tumors, serving as indicators of increased microvessel density (MVD). Radial vessels or perforator vessels are commonly found in lesions with high histological grades of breast cancer or those accompanied by lymph node metastasis [39, 44, 59–61]. In metastatic lymph nodes, heterogeneous enhancement within the lymph nodes is a characteristic feature [44] (Fig. 11). All four subtypes of breast cancer show an increase in area post-enhancement, but the rate of increase in the transverse diameter allows for the prediction of the histological malignancy of malignant tumors [62, 63]. Regarding quantitative parameters, studies have shown that the upward slope serves as the best discriminator for proliferative activity [64].

## CEUS assessment of treatment response to neoadjuvant chemotherapy (NAC)

Patients achieving pathological complete response (pCR) after NAC generally exhibit improved disease-free survival and overall survival compared to non-pCR patients [65]. CEUS enables early prediction of pCR and recurrence-free survival (RFS) in patients with locally advanced breast cancer undergoing NAC therapy by assessing changes in microvascular perfusion [30].

**Fig. 12:**
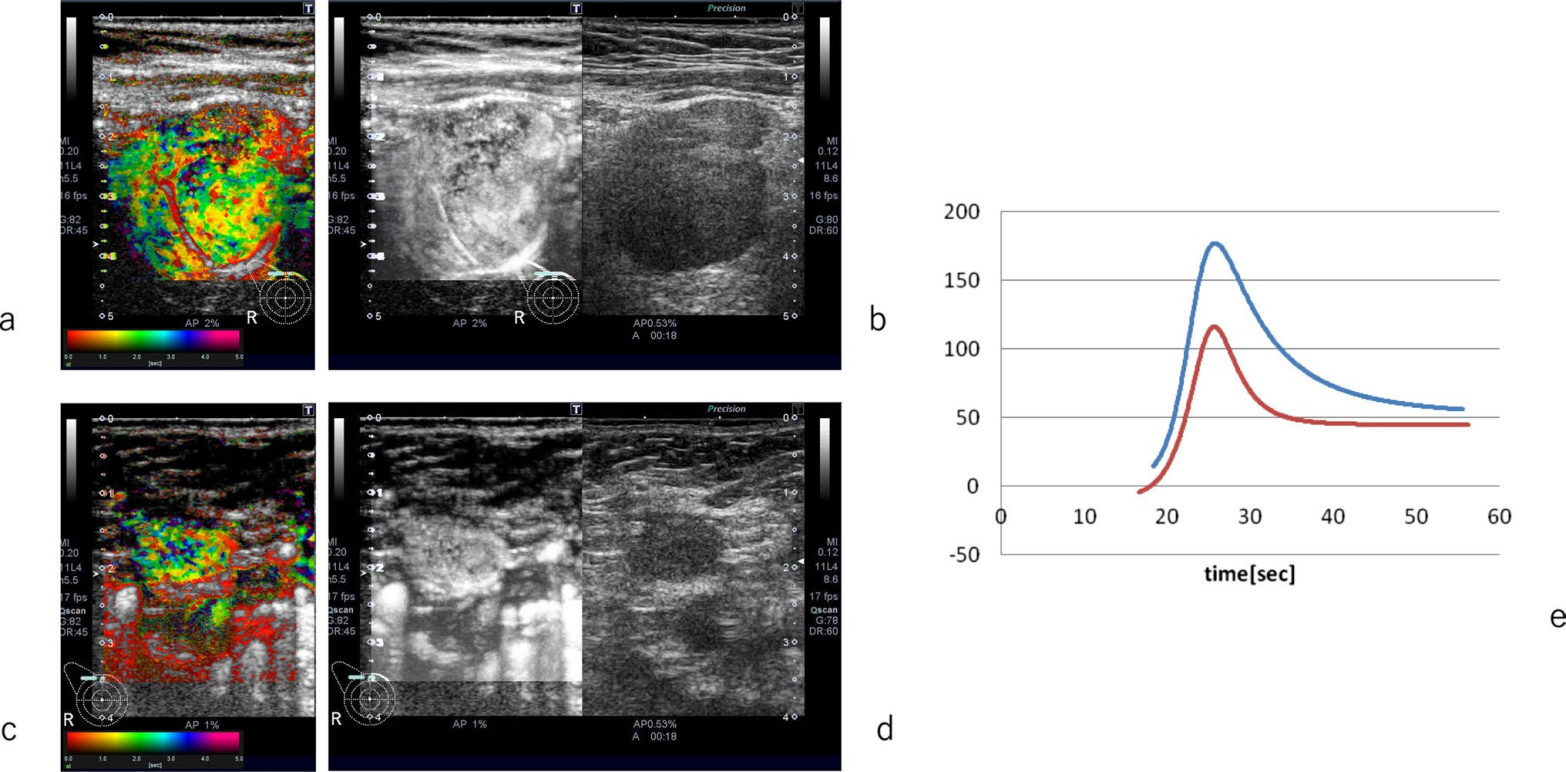
Contrast-enhanced ultrasound (CEUS) before and after neoadjuvant chemotherapy (NAC). **a** Arrival time parametric imaging before NAC. **b** CEUS imaging before NAC. **c** Arrival time parametric imaging after two cycles of NAC. **d** CEUS imaging after two cycles of NAC. e Time-intensity curve before (blue-line) and after NAC (red-line)

CEUS allows for easy dynamic observation and quantification of tumor perfusion [66] (Fig. 12), enabling differentiation between fibrosis and residual tumor after NAC treatment without exposing patients to the risks of radiation [67–73].

Qualitative and quantitative assessment of changes in tumor blood flow during NAC therapy is feasible with CEUS [18–20, 22, 24, 25], and it demonstrates a strong correlation with pathological response outcomes [66–68, 74–79].

The time-to-peak (TTP) on CEUS at the 5th week of NAC is significantly prolonged in responders compared to non-responders, thus serving as a useful tool for evaluation of early response to NAC [67, 80].

Combining conventional US with qualitative CEUS evaluation methods allows for accurate prediction of axillary lymph node status after NAC in breast cancer patients. Studies have shown associations between lymph node medulla boundary, lymph node aspect ratio, CEUS pattern, and post-NAC lymph node pCR [81].

## CEUS for lymph nodes

US evaluation is useful for assessing axillary lymph nodes, but performing CEUS provides more perfusion information, thereby improving the diagnostic accuracy for lymph node metastasis [81–90].

Lymph nodes consist of two vascular systems: lymphatic circulation and blood circulation. In the evaluation of axillary lymph nodes, two CEUS techniques, namely perfusion CEUS and lymphatic CEUS, are utilized.

### Lymphatic CEUS

**Fig. 13:**
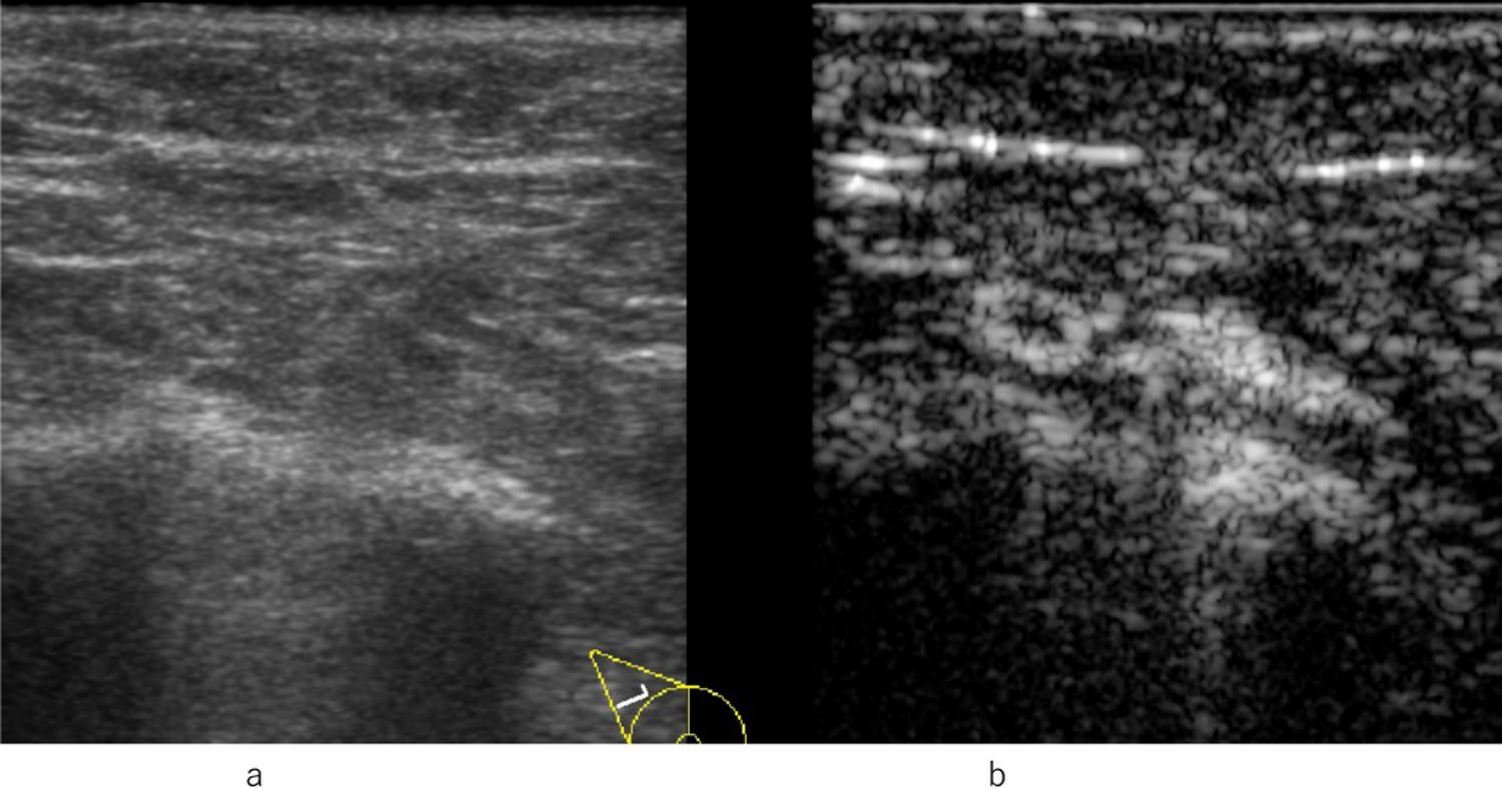
Conventional ultrasound (US) and lymphatic contrast-enhanced ultrasound (CEUS). **a** Conventional US shows a sentinel lymph node (arrow). **b** An oval-shaped contrast-enhanced lymph node (arrow) is visualized within a few minutes after Sonazoid injection

**Fig. 14:**
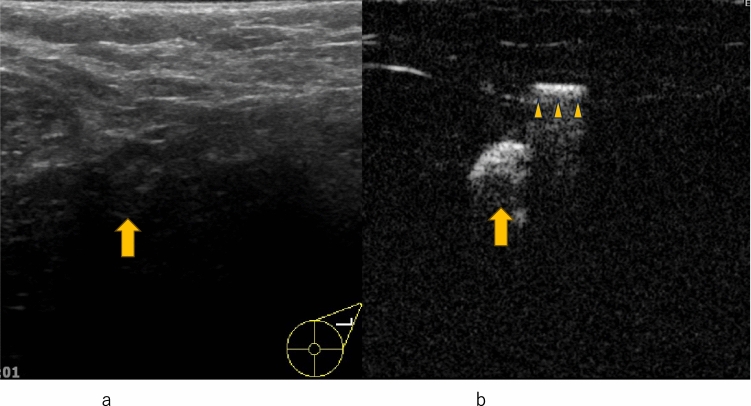
Conventional ultrasound (US) and lymphatic contrast-enhanced ultrasound (CEUS). **a** Conventional US shows a sentinel lymph node (arrow). **b** A lymphatic vessel (arrow head) and an oval-shaped contrast-enhanced lymph node (arrow) are visualized within a few minutes after Sonazoid injection

Lymphatic CEUS identifies sentinel lymph nodes through percutaneous contrast agent administration via lymphatics to diagnose lymph node metastasis. Studies have classified lymph node CEUS enhancement into four enhancement groups, leading to reduced false-positive rates and improved specificity. Niu et al. categorized sentinel lymph nodes into four enhancement patterns, showing that Patterns I & II had a 91.7% negative metastasis rate (Fig. 13, 14), while Patterns III & IV had a higher probability of metastasis [91].

- *Pattern I*: Homogeneous
- *Pattern II*: Featured inhomogeneous
- *Pattern III*: Focal defect
- *Pattern IV*: No enhancement

### Perfusion CEUS

**Fig. 15:**
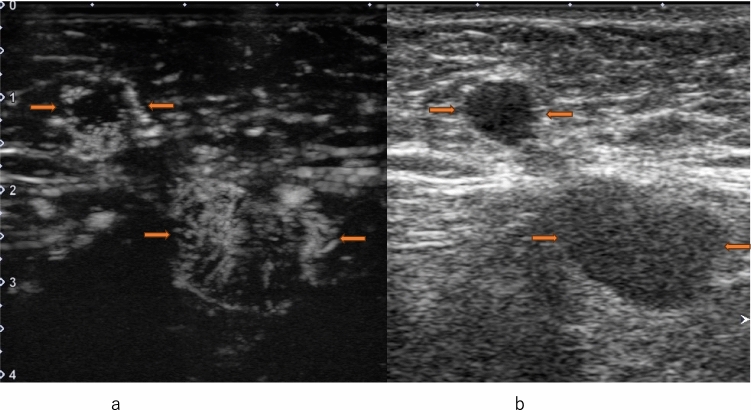
Metastatic lymph node. **a** Contrast-enhanced ultrasound shows heterogeneous enhancement with a clear defect (between arrows). **b** Conventional ultrasound shows an axillary lymph node with cortical thickening (between arrows)

The primary aim of perfusion CEUS is to diagnose lymph node metastasis through contrast agent administration via veins, similar to diagnosing lesions in the breast (Fig. 15). When performing perfusion CEUS after lymphatic CEUS, microbubbles within lymphatic vessels and lymph nodes are shattered using high acoustic pressure before perfusion CEUS is performed. Du et al. concluded that combining conventional US with CEUS enables the appropriate evaluation of axillary lymph nodes.

The arterial phase begins when the contrast agent reaches the lymph nodes, while the venous phase (delayed phase) begins approximately 30–45 s after contrast agent administration [18].

Perfusion CEUS is classified into the following two types:

- *Type A*: Homogeneous enhancement in the arterial phase and homogeneous regression in the venous phase.
- *Type B*: Inhomogeneous regression in the venous phase.

## Discussion

While evaluating minimal blood flow using color Doppler imaging can be challenging [81], CEUS allows for the assessment of blood flow as small as 100 μm [68, 92]. Compared to contrast-enhanced MRI and CT, CEUS offers superior spatial and temporal resolution, enabling real-time observation of tumor microcirculation [25, 45, 93]. Miyamoto et al. evaluated enhancement patterns and found that a diagnosis could be made with the detection of one or a few patterns, demonstrating significantly higher accuracy and specificity with CEUS for lesions both less than 1 cm and over 1 cm in size compared to conventional US and contrast-enhanced MRI [25].

Breast cancer relies on angiogenesis, but the number of vessels, blood flow velocity, and intratumoral vascular resistance do not clearly distinguish malignant from benign breast lesions [94–96]. Angiogenesis is a characteristic pathological process common to most solid tumors, including breast cancer, and is associated with tumor growth, invasion, metastasis, and prognosis [40, 45, 80, 97–104].

The diagnostic accuracy of CEUS varies depending on the size of the lesion [34], which is closely related to vascular density [39, 103, 105–107]. Microvessel density (MVD) and vascular endothelial growth factor (VEGF) expression serve as prognostic markers for breast cancer [108–111].

Benign lesions typically exhibit normal vascular caliber, while in malignant lesions, the distribution of nutrient vessels may be disrupted, sometimes accompanied by arteriovenous shunts [112].

The occurrence rates of high enhancement and heterogeneous enhancement are higher in malignant lesions, whereas low enhancement and homogeneous enhancement are more common in benign lesions. Centripetal enhancement reflects the actual density and distribution of microvessels in malignant lesions [40].

A significant increase in the extent of tumor enhancement after contrast administration is a crucial indicator of malignant lesions and correlates with histopathological findings [40, 64, 113–115]. Additionally, preoperative assessment of tumor spread with CEUS enables accurate evaluation of the extent of resection [113, 116].

While benign tumors mostly exhibit a normal vascular caliber with minimal neovascularization and homogeneous distribution, malignant tumors tend to have more neovascularization and disrupted distribution of nutrient vessels, and may be associated with arteriovenous shunts [40, 112].

Furthermore, in malignant tumors, microvessel density (MVD) and expression of vascular endothelial growth factor (VEGF) tend to concentrate at the tumor periphery [47, 64]. Some low-grade ductal carcinoma in situ (DCIS) may be nourished by normal peripheral vessels without the formation of abnormal peripheral vessels [59, 115, 117–123].

CEUS offers short examination times and can be safely performed in patients with contraindications to gadolinium administration, claustrophobia, or implanted pacemakers. Additionally, it enables more accurate prediction of malignant lesions, thus reducing the need for false-positive biopsies [118, 124, 125].

While benign breast lesions may sometimes yield false-positive CEUS images, this could be attributed to cellular proliferation, hyperplasia, and inflammatory reactions [37, 54, 56, 126–129]. Research on background parenchymal enhancement (BPE) on CEUS has shown similar patterns between patients with malignant tumors and those with benign tumors in each menstrual phase. Cases where BPE exhibits higher enhancement than breast tumors at the peak of contrast are all benign [130].

Although reports suggest that generally similar diagnostic criteria as for tumors can be applied to classifying malignant non-mass abnormalities (NMAs), due to the high proportion of hypoechoic areas in malignant NMAs, current criteria may not be sufficient. It is hoped that future research will elucidate evaluation criteria and diagnostic standards for CEUS based on NMA classification.

Clinical trial results regarding post-NAC treatment in cases where pCR is not achieved are informative [131, 132] as predicting the effectiveness of NAC plays a crucial role in treatment selection, including surgical methods [82].

Due to the difficulty in interpreting extracellular volume changes on DCE-MRI, there is often overestimation or underestimation of the response to NAC [133–135]. Since the contrast agents used in CEUS are present only in the vascular bed, it is possible to obtain results comparable to those of DCE-MRI [67, 73].

Huang et al. performed CEUS before and after two cycles of NAC, but various studies have examined CEUS after the first cycle or after four cycles, etc. [38, 68, 73].

In CEUS studies, various approaches have been taken for setting regions of interest (ROIs), including ROIs encompassing tumor margins, ROIs placed on tumor contours, and ROIs based on hotspots of a certain diameter [75, 112, 136]. Determining appropriate ROIs requires further analysis based on a large number of cases with various ROI settings tailored to the lesion characteristics and objectives.

Regarding sentinel lymph node biopsy using CEUS, Omoto et al. conducted a basic study using a 25% albumin contrast agent in 2002 [137], followed by the first report of identifying sentinel lymph nodes in breast cancer patients in 2006 [138]. Since 2009, evaluation of sentinel lymph nodes using Sonazoid CEUS has been widely performed, with favorable outcomes reported [139–141]. CEUS can provide high diagnostic accuracy in detecting metastatic sentinel lymph nodes, with the risk of metastasis in sentinel lymph nodes showing heterogeneous enhancement being approximately six times higher than those showing homogeneous enhancement [39, 90, 142, 143].

In breast cancer management, diagnosis and treatment of hepatic metastases may be necessary, and CEUS is useful for assessing the efficacy of treatments such as drug therapy due to its simplicity and low patient burden. Evaluation of metastatic liver tumors in the Kupffer phase with Sonazoid CEUS is extremely straightforward and useful [29, 30].

In cases of *BRCA*-positive breast cancer and *BRCA*-positive unaffected individuals, contrast-enhanced MRI is recommended for follow-up, but there are patients who cannot undergo contrast-enhanced MRI [3]. Although data accumulation and analysis are needed in the future, diagnosis with CEUS is considered important as an alternative diagnostic modality to contrast-enhanced MRI.

Regarding the assessment of treatment efficacy for new minimally invasive treatment of breast cancer using microwave ablation, there are reports comparing CEUS and MRI, showing comparable results [144]. US-guided radiofrequency ablation therapy for small breast cancer, which became eligible for insurance coverage in Japan in 2023, may be widely performed [145–148]. Similar to radiofrequency ablation therapy for liver cancer [149–151], CEUS is expected to be used for preoperative diagnosis and assessment of treatment efficacy.

In recent years, diagnosis using artificial intelligence (AI) has been increasingly utilized in various fields [152–154]. In the future, combining CEUS with AI diagnosis is expected to further enhance the diagnostic capabilities for breast cancer.

## Conclusion

For the diagnosis of breast lesions, both qualitative and quantitative evaluations are utilized with CEUS. CEUS has a wide range of applications, including distinguishing between benign and malignant breast lesions, identifying sentinel lymph nodes, diagnosing lymph node metastasis, assessing the efficacy of NAC, and preoperative assessment of breast cancer spread. Additionally, CEUS is a useful diagnostic modality for evaluating treatment efficacy in liver metastases and for treatments such as radiofrequency ablation therapy, with no radiation exposure and minimal patient burden.
